# A nomogram based on peripheral lymphocyte for predicting 8-year survival in patients with prostate cancer: a single-center study using LASSO-cox regression

**DOI:** 10.1186/s12885-024-11929-z

**Published:** 2024-02-23

**Authors:** Jiayi Chen, Feng Yu, Ganyuan He, Wenke Hao, Wenxue Hu

**Affiliations:** Department of Nephrology, Guangdong Provincial People’s Hospital (Guangdong Academy of Medical Sciences), Guangdong Provincial Geriatrics Institute, Southern Medical University, Guangzhou, China

**Keywords:** Peripheral, Lymphocyte, Prognosis, Prostate cancer, Nomogram, Overall survival

## Abstract

**Purpose:**

The purpose of this study was to develop a functional clinical nomogram for predicting 8-year overall survival (OS) of patients with prostate cancer (PCa) primary based on peripheral lymphocyte.

**Patients and methods:**

Using data from a single-institutional registry of 94 patients with PCa in China, this study identified and integrated significant prognostic factors for survival to build a nomogram. The discriminative ability was measured by concordance index (C-index) and ROC curves (Receiver Operating Characteristic Curves). And the predictive accuracy was measured by the calibration curves. Decision curve analyses (DCA) was used to measure the clinical usefulness.

**Results:**

A total of 94 patients were included for analysis. Five independent prognostic factors were identified by LASSO-Cox regression and incorporated into the nomogram: age, the T stage, the absolute counts of peripheral CD3(+)CD4(+) T lymphocytes, CD3(-)CD16(+)CD56(+) NK cells and CD4(+)/CD8(+) ratio. The area under the curve (AUC) values of the predictive model for 5-, 8-, and 10-year overall survival were 0.81, 0.76, and 0.73, respectively. The calibration curves for probability of 5-,8- and 10-year OS showed optimal agreement between nomogram prediction and actual observation. The stratification into different risk groups allowed significant distinction. DCA indicated the good clinical application value of the model.

**Conclusion:**

We developed a novel nomogram that enables personalized prediction of OS for patients diagnosed with PCa. This finding revealed a relative in age and survival rate in PCa, and a more favorable prognosis in patients exhibiting higher levels of CD4 + T, CD4+/CD8 + ratio and CD3(-)CD16(+)CD56(+) NK cells specifically. This clinically applicable prognostic model exhibits promising predictive capabilities, offering valuable support to clinicians in informed decision-making process.

## Introduction

Prostate cancer is indeed a significant health concern, particularly within the context of an aging population. According to the American Cancer Society’s statistics in 2023, the incidence of PCa increased by 3% annually from 2014 to 2019, translating to an additional 99,000 new cases [1]. Therefore, PCa continues to pose a significant social and clinical burden in an aging population.

Prostate-specific antigen (PSA) is a commonly used clinical marker for screening and diagnosis of PCa [2]. After a positive screening test, prognostic biomarkers could provide physicians with improved capabilities to differentiate between PCa that required immediate treatment and those highly unlikely to impact survival so that can be effectively monitored [3, 4]. The clinical stage, PSA level, the Gleason grade, magnetic resonance imaging (MRI), and gene such as PCaA3 and TMPRSS2:ERG have all demonstrated strong associations with the prognosis of PCa patients [5–7]. However, conventional methods of detecting these biomarkers, such as clinicopathological data and gene detection techniques, may have limitations and impose a significant financial burden on patients. Thus, a novel, non-invasive way is needed to identify PCa patients who are at a higher risk of an adverse prognosis. Such an approach would help in developing a precise treatment plan that is tailored to the individual needs of each patients [8]. 

In the process of tumorigenesis, inflammation and immune response have been found to play significant role [9]. Previous evidence suggests that inflammation contributes to the development and progression of various cancers by promoting cancer cell proliferation and metastasis and influencing the response to systemic therapies [10]. Furthermore, research has indicated the association between lymphocyte count and clinical outcomes as well as prognosis in patients with PCa. A reduced lymphocyte count may lead to an ineffective immune response against cancer progression [11]. Lymphocyte subsets were suggested as a key factors potentially in the transition between benign prostate disease (BPD) and PCa [12]. As two subpopulations of CD3 + T lymphocytes, CD8 + T cells exert anti-tumor immune effects through antigen-specific and antigen-nonspecific mechanisms while CD4 + T cells contributing to the activation of CD8 T cells. Data suggest that the higher expression of CD4 and CD8 indicated better overall survival in cancer [13]. Peripheral circulating CD19(+) B lymphocytes, which can be broadly divided into CD19(+)CD5(+) and CD19(+)CD5(-) subsets, are widely recognized as a representative marker of humoral immunity. In PCa patients undergoing carbon ion radiotherapy and experiencing lower grades of side effects, Yang et al. discovered that higher counts of CD19(+) B lymphocytes were associated with better treatment outcome [14]. Natural killer (NK) cells, defined as CD3(-)CD16(+)CD56(+) NK cells, also played critical roles in the immune response against cancer. A study investigating PCa found that patients with both PCa and benign prostatic hyperplasia exhibited decreased levels of NK cells compared to healthy individual [15]. These findings highlighted the potential significance of lymphocyte subsets as prognostic indicators for PCa patients.

The nomogram has been recognized as a reliable tool for quantifying risk by providing numerical probability of clinical events, such as OS [16]. And a number of studies have demonstrated its pivotal role in cancer research: for instance, Liang et al. conducted a comprehensive investigation to identify and consolidate significant prognostic factors, leading to the development of a robust nomogram for accurately predicting survival outcomes in patients with resected non-small-cell lung cancer [17]. Mo et al. innovatively developed functional nomograms to predict specific distant metastatic sites and OS of colorectal cancer patients [18]. Through the combination of clinicopathological features factors for tumor prognosis and a graphical representation of statistical predictive models, PCa nomograms are widely used as prognostic tools [19, 20]. The nomogram can clearly and intuitively quantify the survival probabilities, help to identify patients at different risk, and even provide more precise prediction than the traditional TNM staging systems. Hiremath et al. constructed an comprehensive nomogram combining deep learning-based imaging predictions, PI-RADS scoring, and clinical variables to pertinent clinical variables to discern clinically significant prostate cancer effectively [19]. Meanwhile, Gafita et al. applied LASSO Cox regression to develop externally validated nomograms to predict outcomes after ^177^Lu-PSMA in patients with metastatic castration-resistant PCa [20]. However, there were few nomograms for predicting in the prognosis of PCa with peripheral lymphocyte subsets. Xie et al. showed that drinking, higher PSA level and neutrophil-to-lymphocyte ratio were significant prognostic factors in patients with PCa and established nomograms for 5-year OS and PFS [21]. A retrospective study, with 41 clinic characteristics and the largest sample size of its kind, developed a clinic-ML nomogram which showed that age, B cells (CD3 − CD19+), Neutrophil percentage, PSA and Th/Ts (CD3 + CD4+/CD3 + CD8+) were independent predictors of risk stratifications of PCa patients [22].

There have been limited investigations into the prediction of the long-term survival in PCa by incorporating functional subsets of peripheral lymphocyte subsets into a nomogram. Thus, in this study, the objective was to develop a nomogram for predicting 8-year OS for patients diagnosed with PCa. The construction of this nomogram involved the identification and integration of well-established clinicopathologic variables by LASSO-Cox regression, univariate and multivariate analyses, utilizing an 8-year follow-up dataset derived from a single institutional registry in China. The nomogram was validated and the predictive models’ ability to visualize clinical outcomes was assessed by DCA. In addition, a comparison between the low-risk and high-risk subgroups was conducted to test the discrimination of nomogram.

## Materials and methods

### Study population

A uni-institutional registry of 94 patients with PCa were retrospectively studied in Guangdong Provincial People’s Hospital from January 2001 to June 2021. Patients with a diagnosis of PCa aged ≥ 60 years met the criteria for inclusion. The exclusion criteria included: (1) organ transplantation; (2) infectious diseases; (3) patients on immunosuppressive medications; (4) autoimmune diseases; (5)other active malignant tumors; (6) any condition causing neutropenia and (7) patients without complete survival information (survival months and survival status).

For all patients, clinical data were recorded, including age, PSA, T stage, N stage, M stage, treatment protocols, different T lymphocyte subsets, B lymphocyte subsets and NK cells. All the clinical data were recorded from electronic records in hospital, and laboratory tests were performed in clinical laboratory of Guangdong Provincial People’s Hospital. The study involving human participants was approved by the Ethical Committee of Guangdong Provincial People’s Hospital. Written informed consent was obtained from the patients before the enrollment.

### Follow-up

The primary endpoint of this study was all-cause mortality, and patients’ OS duration was defined as the interval from the date of diagnosis to the date of death or last contact. Follow-up period spanned from January 2001 to June 2021.

### PSA

A photometric method with Cobas C 601 analyzer (ROCHE Diagnostic, USA) was used to detect the serum PSA levels of all patients enrolled in this study.

### Flow cytometry analysis (FCM)

Peripheral blood lymphocyte and lymphocyte subsets were analyzed by flow cytometry. T lymphocytes included the total T lymphocyte, helper T lymphocyte (T helper, CD3(+)CD4(+)) and cytotoxic T lymphocyte (CTL, CD3(+)CD8(+)). B lymphocytes included the total B lymphocyte (CD19(+)), B1 lymphocytes (CD19(+)CD5(+)) and B2 lymphocytes (CD19 (+) CD5(-)). NK cells (CD3(-)CD16(+)CD56(+)) was analyzed as well. Peripheral blood samples were collected in EDTA anticoagulant. The whole blood 100uL were incubated with the following antibodies at 4℃ for 30 min in the dark: CD19-APCa, and CD5-PE. Before staining to each tube, a total of 2 ml of red blood cell lysis buffer was added and incubated for 10 min at room temperature in the dark. The cells were then washed twice with phosphate buffer saline (PBS), and the supernatant was discarded. The cell pellet was dissolved in 300uL of 1% paraformaldehyde. Last, 20,000 cells were acquired by using FACS (BD USA) and were analyzed using Cellquest-Pro analysis software to determine the subpopulation counts.

### Statistical analysis

The statistical analyses were performed using *R* software version 4.2.2. with the followed package: “ggDCA”, “survival”, “rms”, “pROC” and “nomogramFormula”. Normally-distributed variables was expressed as mean ± standard deviation (SD) while categorical data were represented as percentage. Peripheral blood lymphocyte and lymphocyte subsets were transformed into categorical variables based on the outcome using optimal cutoff points determined by the *X*-tile software version 3.6.1 (Yale University, New Haven, USA). And the cutoff points of total CD3(+) T cells, CD3(+)CD4(+) T cells, CD3(+)CD8(+) T cells, the CD4(+)/CD8(+) ratio, total CD19(+) B cell, CD19(+)CD5(+) B cells, CD19(+)CD5(-) B cells, CD3(-)CD16(+)CD56(+) NK cells in patients with PCa from X-tile were 1203.05cells/µL, 365.10cells/µL, 443.45cells/µL, 1.10, 45.83cells/µL, 5.76cells/µL, 35.65cells/µL and 238.87cells/µL, respectively.

The *LASSO* regression model was applied to select the most optimal predictive features in patients with PCa. Cox proportional hazard model was used for univariate and multivariate analyses to find the independent prognostic factors based on the variables selected from *LASSO* regression. Hazard ratio (HR) and 95% confidence intervals were used to measure the impact of each factor on OS. A *P* value < 0.1 was considered as significant. Then the nomogram was formulated on the basis of the results of the multivariate analysis with backward step-down process, employing the Akaike information criterion (AIC) as a stopping rule [23]. Based on the risk score calculated by nomogram, cases were classified as low-risk and high-risk subgroups to test the discrimination of nomogram. The Kaplan-Meier method was utilized for survival analysis to draw the survival curve of each group and the log-rank test was used for statistical analysis to compare their survival time.

The nomogram’s validation consisted of two parts: discrimination and calibration. Discrimination was evaluated using a C-index and ROC curves. The 5-, 8-, and 10-year survival prediction ability of the nomogram were evaluated by ROC curves. The value of the C-index ranged from 0.5 (standing for no discrimination at all) to 1.0 (indicating a perfect discrimination). Calibration was performed by comparing the means of predicted survival with those of actual survival with observed Kaplan-Meier estimates after grouping of the nomogram predicted survival by decile. Additionally, decision curve analysis, a novel tool for evaluating the clinical implementation significance of nomograms, was performed in this study. DCA assesses the predictive models’ ability to visualize clinical outcomes and provides insights for suggesting interventions or treatments for individuals at sufficiently high risk. A *P* value < 0.05 was considered as significant.

## Results

### Patient and demographic characteristics

Among the variables collected in the primary database, Gleason scores and differentiation status were excluded from the analysis due to a high rate of missing values exceeding 20%. A total of 94 patients met the inclusion criteria for this study, with the mean age of 78.7 years (range: 60–94 years). The median follow-up duration of the study was 98 months. The demographic and clinical characteristics of patients included in the analysis are listed in Table 1. The all-cause mortality of 5, 8, and 10 year were 55.3%, 72.3% and 85.1%, respectively.

**Table 1:** Demographic characteristics of patients included in the analysis

Variables	Overall (*n* = 94)
Age, years	78.70 ± 7.76
T stage	
T0-T1	13 (13.8%)
T2-T4	81 (86.2%)
N stage	
N0, Nx	76 (80.9%)
N1	18 (19.1%)
M stage	
M0, Mx	64 (68.1%)
M1	30 (31.9%)
Treatment protocols	
ADT	69 (73.4%)
Others	25 (26.6%)
Prostate-specific antigen, ng/mL	75.3 ± 57.7
Total CD3(+)T lymphocytes, cells/µL	992.09 ± 395.10
CD3(+) CD4(+)T lymphocytes, cells/µL	587.11 ± 245.23
CD3(+) CD8(+)T lymphocytes, cells/µL	346.55 ± 220.04
Total CD19(+)B lymphocytes, cells/µL	154.50 ± 125.58
CD19(+) CD5(+)B lymphocytes, cells/µL	52.60 ± 59.55
CD19(+) CD5(-)B lymphocytes, cells/µL	101.83 ± 80.68
CD3(-)CD16(+)CD56(+)NK cells, cells/µL	333.05 ± 198.92
All-cause mortality	
5 years	52 (55.3%)
8 years	68 (72.3%)
10 years	80 (85.1%)

### Features selection

**Fig. 1:**
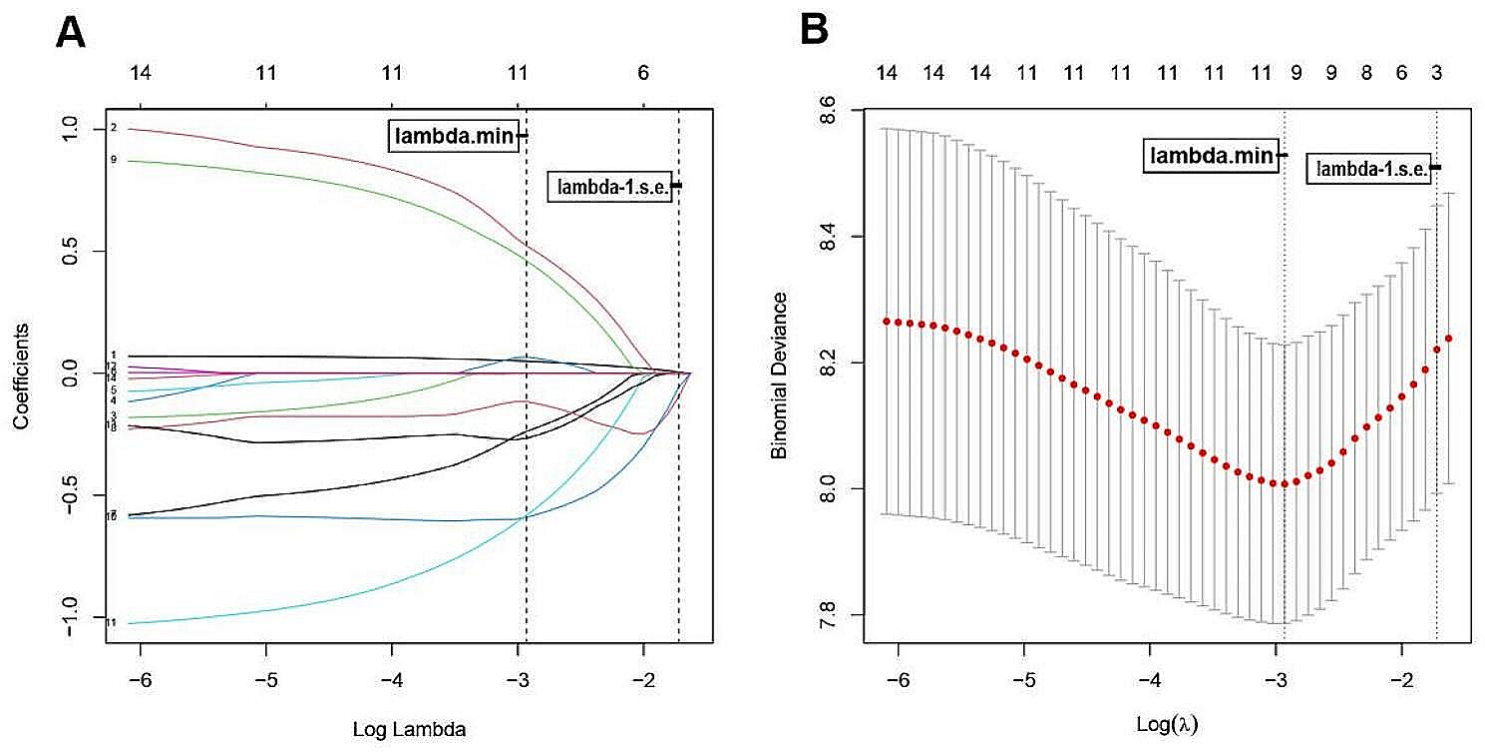
Demographic and clinical features selection using the LASSO regression model. (**A**) LASSO coefficient profiles of the 14 features. A coefficient profile plot was produced against the log (λ) sequence. Vertical line was drawn at the value selected using ten-fold cross-validation. (**B**) Four risk factors selected using LASSO regression analysis. Optimal tuning parameter (λ) selection in the LASSO regression model used ten-fold cross-validation via minimum criteria. The partial likelihood deviance curve was plotted versus log (λ). In both figure, dotted vertical lines were drawn at the optimal values by using the minimum criteria and 1 standard error (SE) of the minimum criteria (the 1-SE criteria). (At minimum criteria including age, T stage, M stage, peripheral CD3(+)T lymphocytes, CD3(+)CD4(+)T lymphocytes, CD19(+)B lymphocytes, CD19(+)CD5(+)B lymphocytes, CD3(-)CD16(+)CD56(+)NK cells and CD4(+)/CD8(+) ratio; At 1-s.e. criteria including age, total CD19(+)B lymphocytes and CD4(+)/CD8(+) ratio.)

The *LASSO Cox* regression model was applied to select the most optimal predictive features. In this study, there were 14 variables for *LASSO* regression analysis: age, T stage, N stage, M stage, treatment protocols, prostate-specific antigen, total CD3(+)T lymphocytes, total CD19(+)B lymphocytes, CD3(-)CD16(+)CD56(+)NK cells, CD4(+)/CD8(+) ratio, CD3(+) CD4(+)T lymphocytes, CD3(+) CD8(+)T lymphocytes, CD19(+) CD5(+)B lymphocytes and CD19(+) CD5(-)B lymphocytes (Fig. 1). The result showed a significant correlation between age, T stage, M stage, peripheral CD3(+)T lymphocytes, CD3(+)CD4(+)T lymphocytes, CD19(+)B lymphocytes, CD19(+)CD5(+)B lymphocytes, CD3(-)CD16(+)CD56(+)NK cells and CD4(+)/CD8(+) ratio and OS at the optimal scores by minimum criteria.

Then, these 9 features at minimum criteria were subjected to univariate cox regression, the results pointed out that age, T stage, M stage, peripheral CD3(+)T lymphocytes, CD3(+)CD4(+)T lymphocytes, CD19(+)B lymphocytes, CD19(+)CD5(+)B lymphocytes, CD3(-)CD16(+)CD56(+)NK cells and CD4(+)/CD8(+) ratio were statistically significant between the two groups (*P* < 0.1). The results of the univariable analysis with variables selected from *LASSO* regression analysis are listed in Table 2. Younger patients exhibited better prognosis. Among all peripheral blood lymphocyte and lymphocyte subsets, higher levels of total CD3(+) T lymphocytes (≥ 1203.05cells/µL vs. < 1203.05cells/µL; *P* = 0.053), CD3(+)CD4(+) T lymphocytes (≥ 365.1cells/µL vs. < 365.1cells/µL; *P* = 0.006), CD4(+)/CD8(+) ratio (≥ 1.10 vs. < 1.10; *P* = 0.004), total CD19(+) B lymphocytes (≥ 45.83cells/µL vs. < 45.83cells/µL; *P* = 0.002) and CD19(+)CD5(+) B lymphocytes(≥ 5.76cells/µL vs. < 5.76cells/µL; *P* = 0.003) were associated with better prognosis. On the contrary, older patients with T2-T4 stage (*P* = 0.065), M1 stage (*P* = 0.024) and higher levels of CD3(-)CD16(+)CD56(+) NK cells (≥ 238.87cells/µL vs. < 238.87cells/µL; *P* = 0.095) showed an unfavorable prognosis in the cohort.

All significant factors in the univariable analysis were entered into multivariable cox regression with backward step-down process. According to the result, age (*P* = 0.003), T stage (*P* = 0.078), the absolute counts of peripheral CD3(+) CD4(+) T lymphocytes (*P* = 0.002), CD4(+)/CD8(+) ratio (*P* = 0.030) and CD3(-)CD16(+)CD56(+) NK cells (*P* = 0.020) were chosen as predictors of patients with PCa (Table 2).

**Table 2:** Prognostic analysis of characteristics and lymphocyte subsets in patients with PCa

Variables	Univariate analysis			Multivariate analysis		
	Hazard Ratio	95% Cl	*P*	Hazard Ratio	95% Cl	*P*
Age, years	1.10	1.04 to 1.17	**< 0.001**	1.10	1.03 to 1.16	**0.003**
T stage						
T0-T1	Reference					
T2-T4	2.45	0.94 to 6.33	**0.065**	1.75	0.91 to 6.43	**0.078**
N stage						
N0, Nx	Reference					
N1	0.95	0.46 to 1.96	**0.881**			
M stage						
M0, Mx	Reference					
M1	2.07	1.10 to 3.89	**0.024**			
Total CD3(+) T lymphocytes, cells/µL						
< 1203.05	Reference					
≥ 1203.05	0.45	0.20 to 1.01	**0.053**			
CD3(+) CD4(+) T lymphocytes, cells/µL						
< 365.1	Reference			Reference		
≥ 365.1	0.36	0.18 to 0.74	**0.006**	0.30	0.14 to 0.65	**0.002**
CD4(+)/CD8(+) ratio						
< 1.10	Reference			Reference		
≥ 1.10	0.39	0.20 to 0.74	**0.004**	0.47	0.24 to 0.93	**0.030**
Total CD19(+) B lymphocytes, cells/µL						
< 45.83	Reference					
≥ 45.83	0.35	0.18 to 0.68	**0.002**			
CD19(+) CD5(+) B lymphocytes, cells/µL						
< 5.76	Reference					
≥ 5.76	0.33	0.15 to 0.68	**0.003**			
CD3(-)CD16(+)CD56(+) NK cells, cells/µL						
< 238.87	Reference			Reference		
≥ 238.87	1.73	0.91 to 3.30	**0.095**	2.29	1.14 to 4.62	**0.020**

### Development of a prognostic nomogram for OS

**Fig. 2:**
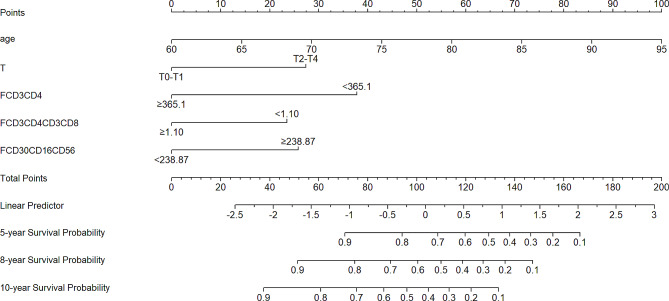
Prognostic nomogram for patients with PCa

A nomogram that incorporated the significant prognostic factors was established with 5-, 8-, 10-year survival probability (Fig. 2). The nomogram illustrated age and the absolute counts of peripheral CD3(+)CD4(+) T lymphocytes as sharing the largest contribution to prognosis, followed by the T stage, absolute counts of peripheral CD3(-)CD16(+)CD56(+) NK cells and CD4(+)/CD8(+) ratio. Each variable was assigned a score on the point scale. By summing up the total score of each variable and locating it on the total point scale, the estimated probability of 5-, 8-, 10-year survival at each time point by drawing a straight line downwards were able to assess.

### The performance of the nomogram for predicting the probability of long-term OS in patients with PCa

**Fig. 3:**
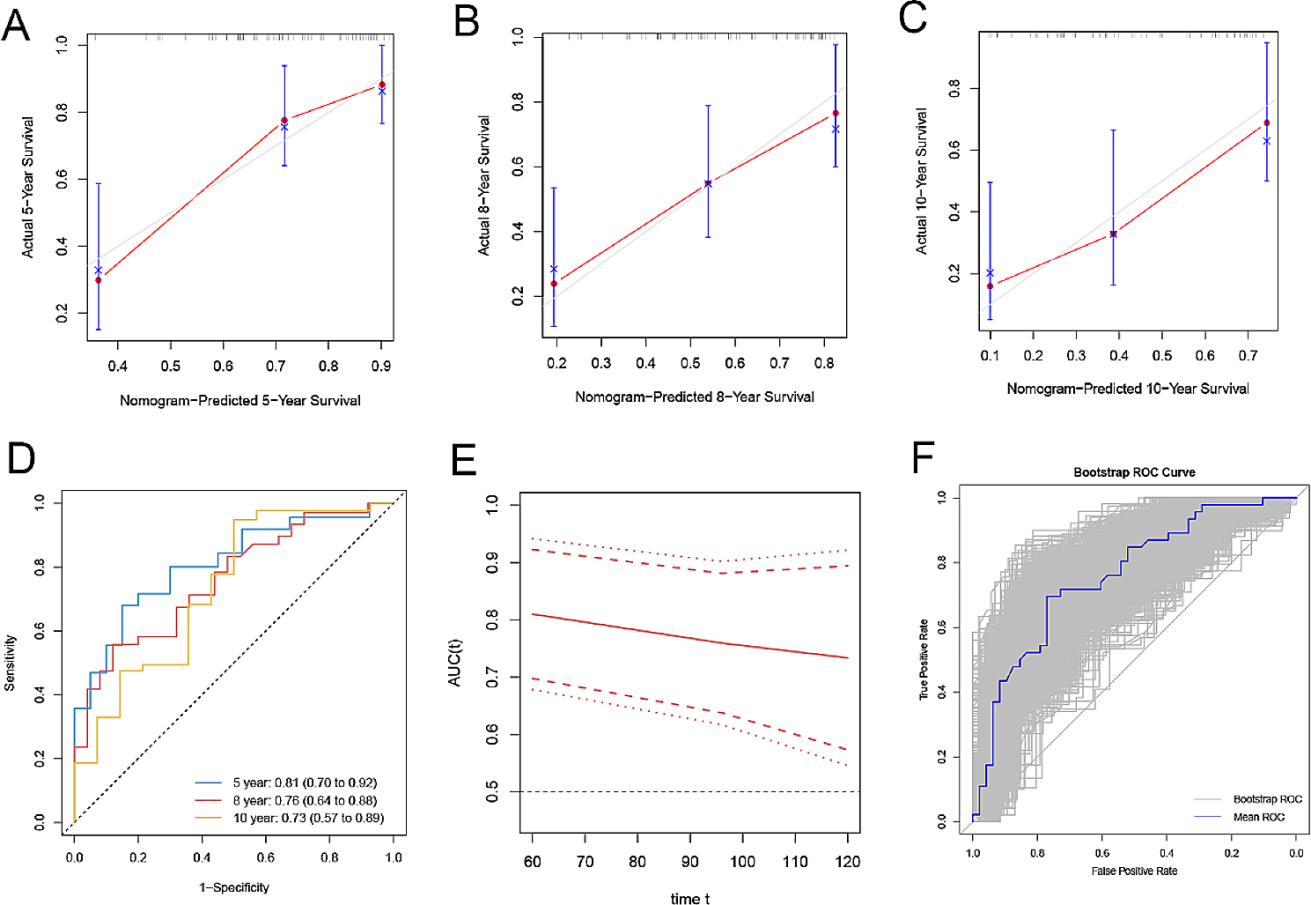
(**A-C**) The calibration curves of the nomogram for predicting 5-, 8-, 10-year (OS) of patients with PCa in the primary cohort. Nomogram-predicted 5-, 8-, 10-year survival is plotted on the x-axis; actual survival is plotted on the y-axis. A plot along the 45-degree line would indicate a perfect calibration model in which the predicted probabilities are identical to the actual outcomes. (**D**) 5-, 8-, and 10-year time-dependent ROC of the model. (**E**) Time-AUC of the model. (**F**) The ROC curve of the internal validation using the bootstrap method (resampling = 1000). Each gray curve in the figure represents the ROC curve of each resampling, and the blue line is the mean ROC curve of the model

The calibration curve of the prediction 5-, 8-, 10-year overall survival nomogram in these patients showed no significant deviation from the reference line, which presented a high degree of credibility (Fig. 3A-C). The AUC values of the predictive model for 5-, 8-, and 10-year overall survival were 0.81 (95% CI, 0.70 to 0.92), 0.76 (95% CI, 0.64 to 0.88), and 0.73 (95% CI, 0.57 to 0.89), respectively (Fig. 3D), confirming the nomogram’s good discrimination to distinguish patients with varying prognosis. The time-AUC (Area Under the Curve) of the model also showed in Fig. 3E. The Harrell’s C-index for the established nomogram to predict the probability of long-term overall survival (OS) in patients with PCa was 0.726 (95% CI: 0.641 to 0.811) and confirmed to be 0.726 (95% CI: 0.656 to 0.825) after adjustment by bootstrapping validation to correct for overoptimism. The AUC value of the predictive model was 0.728 (95% CI, 0.627 to 0.830) and validated to be 0.730 ± 0.051 using the bootstrap method (resampling = 1000). (Fig. 3F).

### Comparison of long-term survival of patients in different risk groups and clinical net benefit of the nomogram

**Fig. 4:**
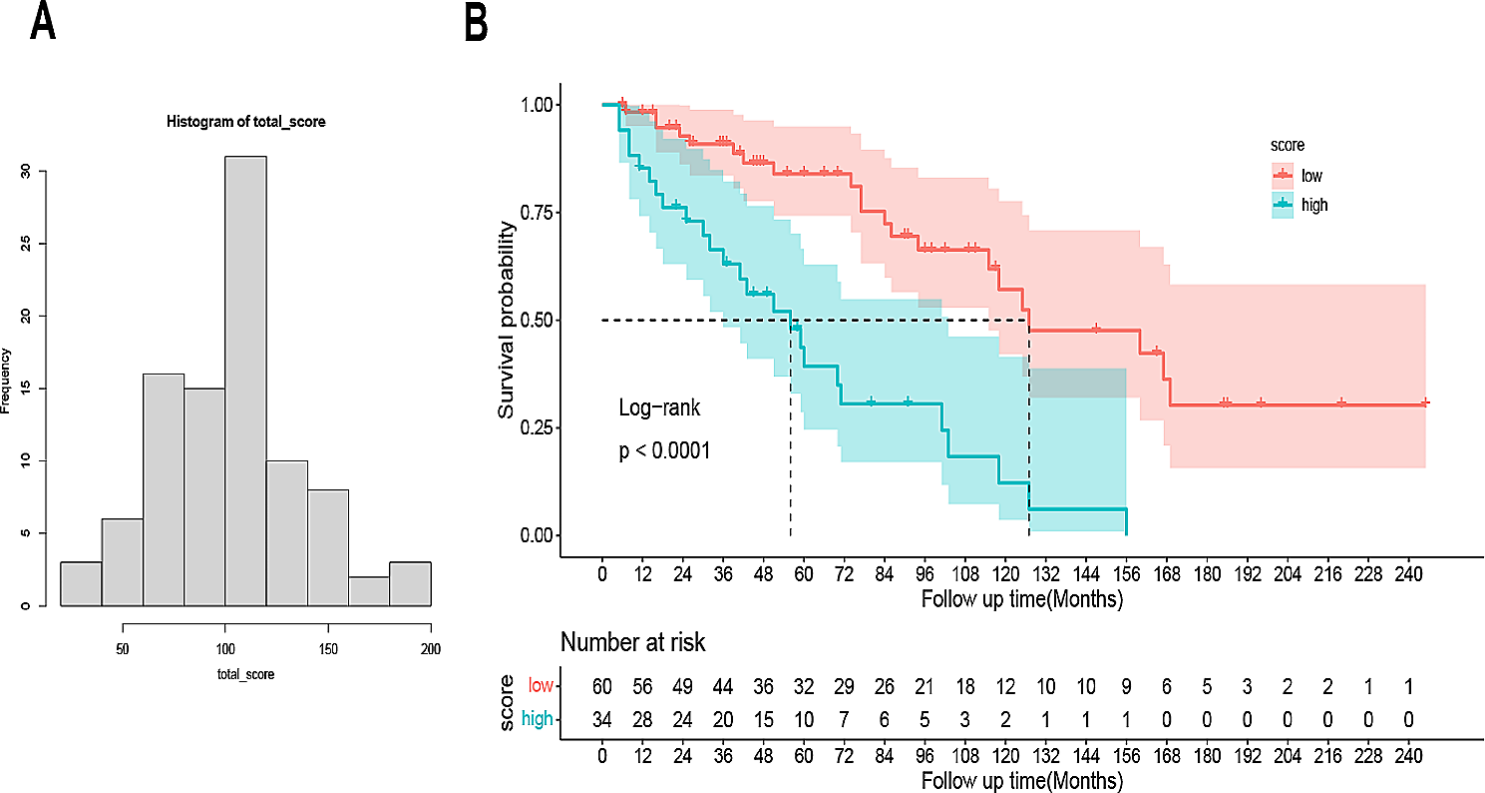
(**A**) The histogram of the total score; (**B**) Overall survival in the subgroup according to a half of the total score

We set a score of 116 as the cutoff value (Youden’s Index) according to the total score calculated by the prognostic nomogram (Fig. 4A), all cases were divided into two subgroups, each of which represented a different prognosis. The prognosis of each subgroup was reflected by Kaplan-Meier survival curve, which is shown in Fig. 4B. Based on OS events, low-risk group (with the total score > 116) had the higher 8-year median OS of than high-risk group (127 months vs. 56 months) with statistically significant distinctions in survival outcomes (*P* < 0.001). We further analyzed the clinic-demographic-immune features of the two groups with distinct prognostic features according to the total score of the nomogram (Table 3). The results showed that patients in high-risk group were older and the proportion of patients in T2-T4 stage were higher. They had lower CD3(+) CD4(+)T lymphocytes, CD4(+)/CD8(+) ratio, total CD19(+)B lymphocytes, CD19(+) CD5(+)B lymphocytes, CD19(+) CD5(-)B lymphocytes and higher CD3(-)CD16(+)CD56(+)NK cells compared to those in low-risk group (*P* < 0.05).

**Table 3:** Demographic characteristics of the two groups with distinct prognostic features

Variables	Overall (*n* = 94)	Low-risk group (*n* = 60)	High-risk group (*n* = 34)	*P* value
Age, years	78.70 ± 7.76	76.0 ± 7.60	83.5 ± 5.47	<0.001
T stage				0.028
T0-T1	13 (13.8%)	12 (20.0%)	1 (2.94%)	
T2-T4	81 (86.2%)	48 (80.0%)	33 (97.1%)	
N stage				0.589
N0, Nx	76 (80.9%)	50 (83.3%)	26 (76.5%)	
N1	18 (19.1%)	10 (16.7%)	8 (23.5%)	
M stage				1.000
M0, Mx	64 (68.1%)	41 (68.3%)	23 (67.6%)	
M1	30 (31.9%)	19 (31.7%)	11 (32.4%)	
Treatment protocols				0.454
ADT	69 (73.4%)	42 (70.0%)	27 (79.4%)	
Others	25 (26.6%)	18 (30.0%)	7 (20.6%)	
Prostate-specific antigen, ng/mL	75.3 ± 57.7	75.6 ± 56.7	74.7 ± 60.3	0.945
Total CD3(+)T lymphocytes, cells/µL				0.159
< 1203.05	71 (75.5%)	42 (70.0%)	29 (85.3%)	
≥ 1203.05	23 (24.5%)	18 (30.0%)	5 (14.7%)	
CD3(+) CD4(+)T lymphocytes, cells/µL				<0.001
< 365.10	16 (17.0%)	3 (5.00%)	13 (38.2%)	
≥ 365.10	78 (83.0%)	57 (95.0%)	21 (61.8%)	
CD3(+) CD8(+)T lymphocytes, cells/µL				0.963
< 443.45	68 (72.3%)	44 (73.3%)	24 (70.6%)	
≥ 443.45	26 (27.7%)	16 (26.7%)	10 (29.4%)	
CD4(+)/CD8(+) ratio				<0.001
< 1.10	17 (18.1%)	4 (6.67%)	13 (38.2%)	
≥ 1.10	77 (81.9%)	56 (93.3%)	21 (61.8%)	
Total CD19(+)B lymphocytes, cells/µL				<0.001
< 45.83	15 (16.0%)	2 (3.33%)	13 (38.2%)	
≥ 45.83	79 (84.0%)	58 (96.7%)	21 (61.8%)	
CD19(+) CD5(+)B lymphocytes, cells/µL				<0.001
< 5.76	10 (10.6%)	1 (1.67%)	9 (26.5%)	
≥ 5.76	84 (89.4%)	59 (98.3%)	25 (73.5%)	
CD19(+) CD5(-)B lymphocytes, cells/µL				<0.001
< 35.65	15 (16.0%)	2 (3.33%)	13 (38.2%)	
≥ 35.65	79 (84.0%)	58 (96.7%)	21 (61.8%)	
CD3(-)CD16(+)CD56(+)NK cells, cells/µL				0.015
< 238.87	36 (38.3%)	29 (48.3%)	7 (20.6%)	
≥ 238.87	58 (61.7%)	31 (51.7%)	27 (79.4%)	

### Clinical usefulness of the predictive nomogram

**Fig. 5:**
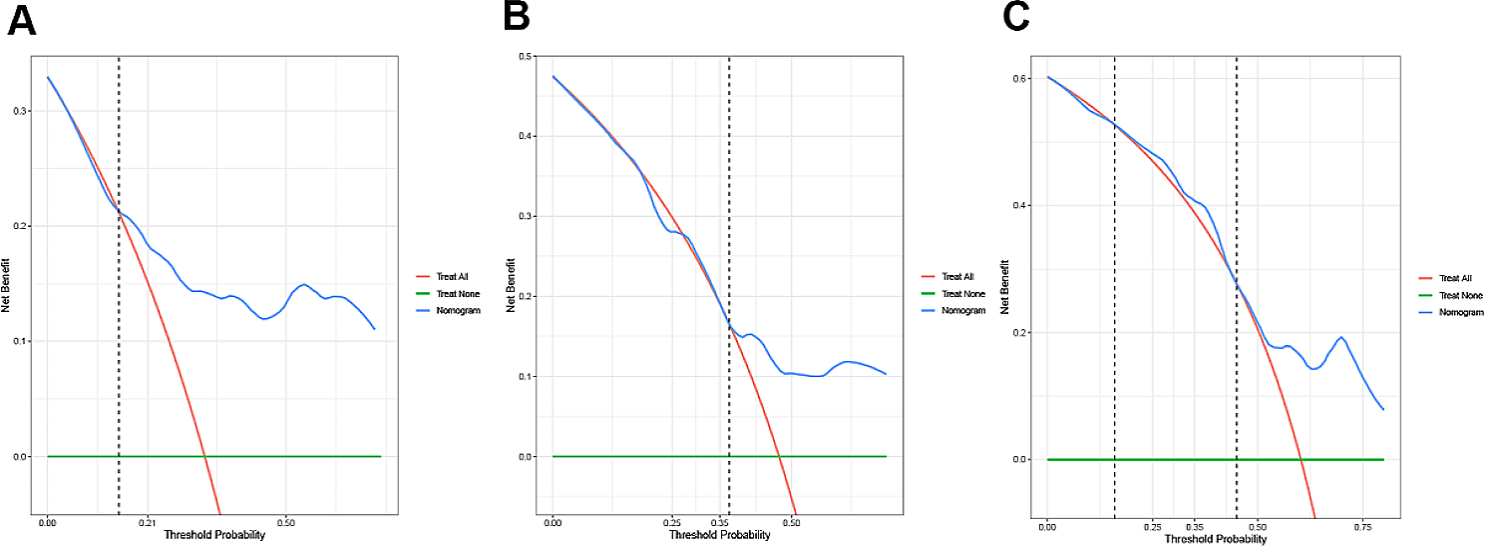
Decision curve analyses with clinical net benefits of the nomogram at 5 year (**A**), 8 year (**B**) and 10 year (**C**)

DCA was used to appraise the clinical usefulness of the predictive nomogram. In the prognostic models, determining a clinically useful range for net benefit is still a topic of debate and lacks consensus [24]. In clinical practice, applying the nomogram to would be advantageous when the threshold probabilities were higher than 10% at least. From the perspective of the decision curve in the study (Fig. 5), if the threshold probability of a patient was higher than 0.15 for 5-year OS, or higher than 0.37 for 8-year OS, or higher than 0.45 for 10-year OS, respectively, using this nomogram to predict the long-time survival of patients with PCa would achieve a favorable net benefit with good clinical implementation significance. The 10-year risk model also improved clinical decision making when the threshold probability of a patient was between 0.16 and 0.45.

## Discussion

Despite several previously reported prognostic models, a nomogram has not been developed for long-time survival of PCa [22, 25]. Thus, we sought to develop a clinical nomogram for predicting long-term survival of patients with PCa based on the absolute counts of peripheral lymphocyte subsets. A total of 94 PCa patients were included in the retrospective study. Specifically, patients aged over 60 years old were enrolled, providing valuable prognostic insights for older individuals with PCa. Importantly, this study had a prolonged follow-up duration ranging from 5 to 245 months (with a median follow-up duration of 98 months), which yielded substantial evidence regarding long-term clinical outcomes.

Through LASSO regression, univariable analysis and subsequent multivariable analysis, we successfully identified age, the T stage, the absolute counts of peripheral CD3(+) CD4(+) T lymphocytes, CD4(+)/CD8(+) ratio and CD3(-)CD16(+)CD56(+) NK cells as significant prognostic factors. These findings were in high concordance with previous reports on risk factors for PCa [14]. The nomogram that incorporated the factors above was established with 5-, 8-, 10-year survival probability, and illustrated the largest contribution of age as well as the absolute counts of peripheral CD3(+)CD4(+) T lymphocytes. Meanwhile, the 5-, 8-, 10-year median OS was distinguishable between the two risk stratification divided by the nomogram score. Thus, the simple nomogram can serve as an insight tool for assessment and surveillance of clinical prognosis of PCa.

The nomogram analysis indicated that patients with PCa who were younger and had higher levels of CD3(+) CD4(+) T lymphocytes and CD4(+)/CD8(+) ratio exhibited longer survival. Similar findings have been reported in other studies investigating cancer progression [12, 13, 26, 27]. The presence of CD3(+)CD8(+) T cells did not appear to be associated with the survival of PCa patients, which might be attributed to their role as effector cells within the tumor microenvironment [27]. On the other hand, CD3(+)CD4(+) T cells play a crucial role in antitumor immunity by acting as “helper” cells in cell-mediated immune responses against tumors [28]. These circulating CD4(+) T cells contribute to the activation of peripheral blood CD8(+) T cells within the tumor microenvironment through the production of IL-21, thereby enhancing the cytotoxic function of infiltrating CD8(+) T cells. This mechanism helps control chronic viral infections and tumor progression [29]. Furthermore, the capacity of immune system in PCa patients to react against pathogens had reduced when the CD4(+)/CD8(+) ratio inverted. On the contrary, the cohort study revealed that high levels of CD3(-)CD16(+)CD56(+) NK cells were associated with a worse long-term clinical outcome in PCa patients. Previous research has highlighted the anti-tumor activities of NK cells. Mao et al. found that a low level of NK cells was an unfavorable prognostic factor for PCa patients [26]. This could be attributed to the protective effect of NK cells against tumor progression, especially in cases where the tumor is difficult to control [30]. The differences observed may be partially explained by the presence of a considerable proportion of non-functional NK cells mixed in peripheral blood. Overall, this data serve as a reminder that the relative changes of T cells and NK cells influence the long-term prognosis of PCa. By summing up the total score of each variable and locating it on the total point scale, the estimated probability of 8-year survival at each time point by drawing a straight line downwards on the nomogram were able to assess.

Validation of the nomogram is crucial to prevent model overfitting and assess its generalizability [31]. In this current study, the calibration curve of the prediction 5-, 8-, and 10-year overall survival demonstrated excellent concordance between predicted probabilities and actual observations, ensuring the repeatability and reliability of the developed nomogram. The high AUC values and Harrell’s C-index of the predictive model for 5-, 8-, and 10-year overall survival provide strong evidence of the nomogram’s excellent discriminatory ability to distinguish patients with varying prognosis.

The application of nomograms in clinic diagnosis has gained popularity in recent years due to their simplicity, intuition, and interpretability [17, 32]. To the best of our knowledge, this is the novel nomogram developed for long-term follow-up that predicts the individual prognosis of patients with PCa based on peripheral lymphocyte count. By incorporating the most significant examination feature as variables in the nomogram, both physicians and patients can benefit from personalized survival predictions. Moreover, the identification of distinct risk subgroups among patients may significantly influence treatment decisions and care options. The established nomogram was considered a precise prognostic model.

However, this study also has some limitations. Firstly, this was a retrospective study with parameters missing, such as pathological features (Some elderly patients are clinically diagnosed) and therapy information except for surgery. Secondly, the underlying mechanism of different lymphocyte subsets in PCa patients was not investigated in the study. More frequent monitor and repeated measurements are needed to assess the accuracy of long-term outcome predictions. Thirdly, the sample size in this study was not large enough. Therefore, further studies and continued patient follow-up are needed to evaluate and improve the utility and applicability of the model.

## Conclusion

In conclusion, we applied LASSO-Cox regression to develop and validate a new nomogram to predict the survival of patients with PCa. This finding revealed the relative in age, peripheral lymphocyte count and survival rate in PCa. The combination of age, the T stage, peripheral CD3(+) CD4(+) T lymphocytes, CD4(+)/CD8(+) ratio and CD3(-)CD16(+)CD56(+) NK cells, possessed a high diagnostic efficiency in the 8-year OS of patients with PCa. This straightforward nomogram, characterized by enough discrimination, calibration and good clinical applicability, empowers clinicians to make precise estimations of individual patient survival and make more informed decisions regarding treatment strategy.

## Data Availability

The datasets generated and analysed during the current study are available from the corresponding author on reasonable request.
